# Adolescents’ Interactions with Their Food Environment in Vietnam: A Participatory Video-Based Study on Factors Shaping Dietary Behavior

**DOI:** 10.1016/j.cdnut.2026.107706

**Published:** 2026-04-25

**Authors:** Deborah Nabuuma, Brice Even, Hang Thi Minh Thai, Thi Thanh Thu Pham, Andrea Fongar-Tiralla, Tuyet Mai Truong, Irmgard Jordan, Céline Termote

**Affiliations:** 1 Department of Food Environment & Consumer Behaviour, Bioversity International, Penang, Malaysia; 2 Department of Food Environment & Consumer Behaviour, International Center for Tropical Agriculture, Hanoi, Vietnam; 3 Department of Food Environment & Consumer Behaviour, Bioversity International, Rome, Italy; 4 Vietnam National Institute of Nutrition, Hanoi, Vietnam; 5 Department of Food Environment & Consumer Behaviour, Bioversity International, Nairobi, Kenya

**Keywords:** food environment, adolescents, Vietnam, participatory videomaking, nutrition, food choices

## Abstract

**Background:**

Adolescence is a critical life stage, marked by increasing personal autonomy and peer influence. Food environments encompass the availability, accessibility, and appeal of food options. Exposure to food environments dominated by unhealthy and unsustainable food options can reinforce poor dietary habits with lasting impacts on adolescents’ nutrition and health. In rapidly changing food environments, as in Vietnam, it is critical to understand how adolescents navigate their food environments.

**Objectives:**

This study used a participatory action-research videomaking approach to explore how adolescents in Vietnam perceive and interact with their food environment.

**Methods:**

In total, 52 adolescents (26 boys and 26 girls) aged 15 to 17 y from urban, peri-urban, and rural settings participated to create 7 interactive videos portraying their immediate food environments. Content analysis of the videos and the workshop transcripts was conducted, combining deductive and inductive coding in MAXQDA 24 data analysis software.

**Results:**

Adolescents reported a diverse range of food outlets near their homes and schools, supplying a combination of fresh, locally sourced foods alongside packaged, processed, and ultraprocessed foods. Their choices of food outlet and product were shaped primarily by affordability, outlet proximity, and time constraints, with additional influences shaped by social media, taste, trendiness, novelty, popularity, and familiarity.

**Conclusions:**

Our findings underscore the importance of adopting multilevel strategies that enhance the accessibility, affordability, appeal, and convenience of nutritious foods, particularly near schools. Strategies should include strengthening adolescents’ capacity to understand food labels and critically evaluate food marketing to make informed decisions, and regulating the marketing of unhealthy food products, particularly in and around schools and on digital platforms. Empowering adolescents as active participants in shaping their food environments offers promising pathways toward improved diet quality and nutrition outcomes. Further research is needed to better understand the complex and rapidly changing food landscapes that Vietnamese adolescents navigate.

## Introduction

Diets are rapidly evolving worldwide, closely linked to food systems transformations, driven by urbanization, rising incomes, supply chain globalization and modernization, technological developments in food production and processing, and shifts in public policy [[1], [2], [3], [4]]. Healthy and sustainable diets are still unaffordable to about one-third of people worldwide, and three-quarters of the global food-insecure population lives in urban and peri-urban areas [5,6]. Healthy diets are characterized by 4 principles: adequacy, balance, diversity, and moderation [7]. Sustainable diets encompass these characteristics while additionally emphasizing broader dimensions, including cultural acceptability, accessibility, economic fairness and affordability, nutritional adequacy, safety and healthfulness, as well as protection of biodiversity and ecosystems and the responsible use of natural and human resources [8]. The food environment—the interface where people interact with food systems to make food choices—is increasingly recognized as a critical intervention space for achieving healthier and more sustainable diets for everyone [9].

Globally, 42% of the population is aged <25 y, comprising 1.2 billion adolescents aged 10 to 19 y, most of whom (90%) live in low- and middle-income countries (LMICs) [10,11]. Adolescents represent an important group for dietary interventions, as this life stage is marked by increasing autonomy, peer influence, and greater exposure and interactions with food vendors and messaging [11]. Dietary habits formed during this period often persist through adulthood, with long-term consequences for health and well-being [12]. Evidence drawn from global school-based student health surveys in Africa, Asia, Oceania, and Latin America, conducted between 2008 and 2015, shows that adolescents frequently consume fast food and sugar-sweetened beverages, with 46% consuming fast food at least once per week and 43% consuming carbonated soft drinks on a daily basis [13]. At the same time, fruit and vegetable consumption remains low in most countries [14]. Changes in their food environment have profound and lasting impacts on adolescents’ dietary behavior and overall health outcomes [14,15]. Global trends highlighted by Beal et al. [13] emphasize the importance of examining how local food environments shape adolescent diets in rapidly developing contexts such as Vietnam.

The risk of malnutrition among adolescents in Vietnam is increasing [16,17]. A steady rise in overweight and obesity among adolescents in Vietnam was reported at 19% and 8%, respectively, in 2020 among children aged 5 to 19 y [18]. This has been notably linked to the shift from traditional diets toward increased consumption of animal products, processed, and convenience foods, as adolescents struggle with balancing hedonistic (driven by pleasure) and healthy eating values, and weigh immediate gratification against longer-term consequences [19]. Evidence shows declining vegetable consumption alongside wider availability of sweets and sugary drinks, and greater reliance on out-of-home food by adolescents and adults [[20], [21], [22], [23]]. These shifts in Vietnam are shaped by multiple interacting drivers. On the supply side, the expanding availability, accessibility, and affordability of diverse food products have reshaped consumer options and incentives [20]. On the demand side, rising expectations for convenience and novelty further stimulate consumption and encourage manufacturers and vendors to respond with new products [23,24]. Evidence shows that adolescents and children play an increasingly significant role in household food choices through “pester power,” as parents are increasingly pressured into considering their tastes and preferences when planning meals [22,23].

Schools have been identified as key entry points for addressing adolescents’ nutrition knowledge gaps [25]. However, knowledge alone does not necessarily translate into the consumption of healthier foods, and more systemic approaches addressing several domains of food environments are more likely to support the adoption of healthy diets [26,27]. Such approaches require recognizing adolescents’ values, preferences, and practices beyond nutrition or health, and a consideration of how they interact with their (changing) food environment. A recent scoping review highlighted that adolescents are engaging with food environments in multiple ways, particularly food provision and retail, confirming their potential to contribute actively and meaningfully to food environment transformation [28].

This study explores how adolescents in Vietnam perceive and interact with their food environments through a participatory videomaking process, examining where, when, and how adolescents obtain food and how they perceive the actors, food, places, and processes that shape their food experiences and choices. Participatory videomaking, which is rooted in participatory action research, provides both a process and a medium for participants to reflect on their lived experiences, cocreate knowledge, and communicate with wider audiences [[29], [30], [31]]. It has been applied for knowledge creation and learning, and can foster social change, capacity development, and people-centered advocacy [[29], [30], [31]]. Involving adolescents as active partners in such cocreation processes is critical to shaping food environments that enable affordable, culturally acceptable, and healthy diets [14,24,[32], [33], [34]]. Adolescents are the consumers of today and can act as catalysts for change, advocating for healthier eating habits and influencing family food choices. By using a participatory videomaking approach, participants were directly involved in producing visual data, exercising control over what was filmed and how their experiences were represented, thereby not only documenting adolescents’ lived experiences but also enabling them to interpret and communicate the opportunities and challenges they face. The study thus contributes new evidence on the evolving food environments of Vietnamese adolescents and the potential for youth-led engagement to inform healthier and more equitable food systems. These insights provide a compelling basis for policy and program interventions that reflect adolescents’ realities and perspectives.

## Methods

### Food environment conceptual framework

**FIGURE 1:**
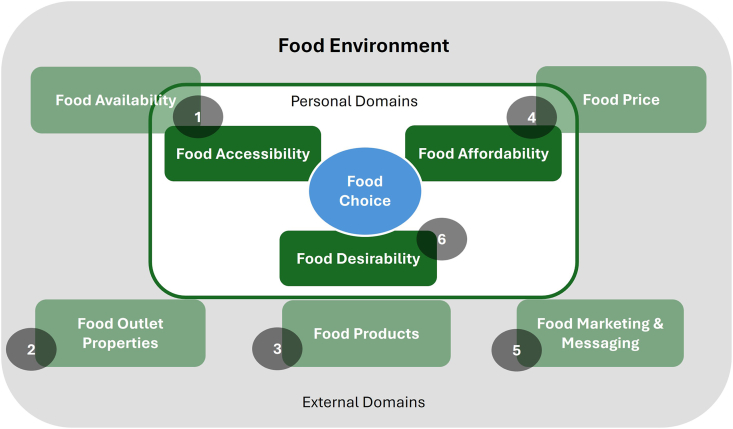
The study’s food environment conceptual framework, adapted from Turner et al. [9], showing the 6 “core codes” corresponding to the 6 food environment domains.

Food environments are the physical, economic, political, and sociocultural contexts in which people engage with the food system and that shape their decisions about acquiring, preparing, and consuming food [35]. This study adapts the conceptual framework and looks at adolescent food environments through 6 domains that influence food choices: *1*) food availability and accessibility; *2*) food price and affordability; *3*) food outlet properties; *4*) food products; *5*) food marketing and messaging; and *6*) food desirability (Figure 1) [9]. This framework was selected given the clear distinction between external and personal food environments that enabled accessible linkage between food environments and consumption, particularly for participants with no prior familiarity with food environment terminology or categorical frameworks. The domains of food availability and accessibility were combined, as both are interconnected. Availability refers to the presence (or absence) of a food source or product, whereas accessibility is relative to an individual’s ability to access that product and is highly dynamic, according to individual context. Likewise, price and affordability have been combined as both determine economic access to food. Food prices and an individual’s available financial resources affect how an individual perceives affordability. Vendor and product properties, on the other hand, were disaggregated into 2 domains. Lastly, the food desirability domain refers to the individual’s perception of food, including personal preferences, acceptability, tastes, desires, attitudes, culture, and the knowledge and skills required to understand, select, and prepare food.

### Study sites and sample

The participatory action-research study was conducted from June to December 2023, in 3 districts of northern Vietnam: Moc Chau (rural site), Dong Anh (peri-urban site), and Dong Da (urban site). The rural site, located in the mountainous province of Son La, is characterized by a food system that is primarily described as “traditional,” but that shows clear signs of modernization [36]. The peri-urban site, within Hanoi municipality, is marked by rapid urbanization, intensive crop and livestock production, and has a high percentage of migrants and commuting workers [37]. The urban site, also within Hanoi municipality, is characterized by a high population density and a wide range of both formal and informal food retail outlets [38].

The mobilization of adolescents for this study was coordinated by the Vietnam National Institute of Nutrition in collaboration with local authorities and the research team. At each site, the study team, through the selected high schools in the rural and peri-urban sites and through a community health center in the urban site, identified and invited adolescents aged 15 to 17 y, preferably in first-year senior secondary school (years 10 and 11), to join the study. This age group was selected based on the cohort’s mobility and the autonomy necessary for the study, while avoiding interference with the exam preparation among older adolescents (>17 y). In total, 52 adolescents (26 boys and 26 girls; mean age of 15.6 y) participated in the study (9 boys and 9 girls in the rural and peri-urban sites and 8 boys and 8 girls in the urban site). The sample size at each site was determined to align with the participatory video methodology and the practical requirements of the study design, balancing opportunities for engagement and collaboration with the available resources and capacity to support participants throughout the video production process. Written informed consent was obtained from the adolescents’ parents or guardians, and written informed assent was obtained from all participating adolescents. All invited adolescents provided parental consent and adolescent assent, except for 1 male participant in the urban site, who declined and was replaced. No participants withdrew during the study.

### Participatory videomaking process

**FIGURE 2:**
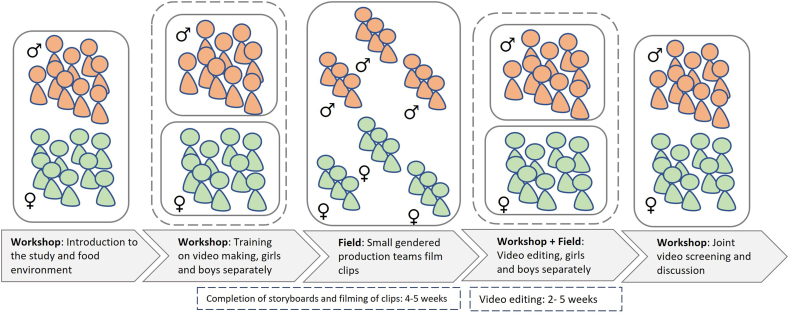
The participatory videomaking process conducted in each study site.

The participatory video production stage followed a 5-step process (Figure 2). Steps 1 and 2 consisted of participatory workshops to introduce the study objectives and the concept of food environments, and to train participants on videomaking using smartphones and storyboard development, as well as ethical and safety considerations during filming. In step 3, the girls and boys worked separately to prepare and finalize the storyboards for their videos. Gender segregated video production was adopted to create more inclusive spaces for expression, increase equity in participation, and reduce any potential power imbalances. They formed gender-separated “production” teams (*n* = 3 per sex) and proceeded to capture video footage of their food environment, including food outlets (where food is obtained and/or consumed), food products, food prices, food messaging (marketing), and their views and food-choice motivations. They recorded meals on both school and nonschool days, according to their own schedules, autonomously choosing what aspects of their meals they wanted to document. Participants received continuous (on-demand) technical support from the research team and were observed (without intervention) by the research team throughout the whole process, as per the observation protocol developed by the research team (mainly capturing group dynamics and any challenges arising). Step 4 involved a practical workshop to support the video editing process. The production teams regrouped by gender to agree on a collective story, selecting, cutting, and sequencing the video clips. The video editing continued after the workshops, with participants finalizing the cutting and adding a variety of features to enhance the storytelling and visual appeal (such as captions, voice-overs, visual and sound effects, and background music). In step 5, participants screened and jointly evaluated the videos. They presented their work, discussed the food environment perspectives depicted in the videos, evaluated how well the videos reflected adolescents’ lived realities in their community, and identified any key missing elements. The mixed-gender screenings were conducted to support cross-gender dialogue and collective reflection on the food environments and dietary choices. The workshops were conducted at the selected high schools in the rural and peri-urban sites and the community health center in the urban site. A representative from each institution was present during all workshops, primarily to provide logistical support, and did not participate in the research activities.

The participatory video methodology guided the methodological choices, positioning adolescents as active contributors who determined the focus, content, and framing of their videos, whereas researchers maintained a facilitative role limited to technical training, logistical coordination, and supportive discussion. The workshops were designed to extend participation beyond filming by enabling collaborative reflection and evaluation of the videos. In addition to the voluntary participation consent, attention to power dynamics and ethical practice included recruitment and activity scheduling that did not conflict with school and student commitments, as well as rapport-building and facilitation strategies that encouraged open dialogue.

### Data collection and analysis

Qualitative data consisted of the workshop transcripts and the final videos. The workshop discussions were audio-recorded, transcribed in Vietnamese, translated into English, and validated by the Vietnamese-speaking research team members. In total, we obtained 9 workshop transcripts (3 sites and 3 workshops per site) and 7 videos. The project design had foreseen 2 videos per site (1 by the boys and 1 by the girls). However, in the urban site, the boys’ group split into 32 teams (preferring to work as 2 groups as per their interpersonal group dynamics), resulting in 4 boys’ videos and 3 girls’ videos. The video audio tracks were translated into English, and subtitles were added to create English versions; both the audio and the visual content were analyzed. The visual, verbal, and nonverbal elements in the videos relevant to the research questions were noted in analytic memos, coded, and analyzed. Where both verbal narration and visual imagery were present, they were treated as complementary sources of meaning. For the transcripts, we only analyzed the segments relevant to the food environments and adolescents’ perceptions, excluding the facilitator presentations and technical training on video production. A content analysis, which combined deductive and inductive elements, was applied using 3 basic operations by 4 research team members (2 junior and 2 senior researchers) [39]. A preliminary coding exercise used a semideductive approach based on the 6 domains of the food environment conceptual framework (Figure 1), which served as the basis for top-level coding categories. Although the overarching structure was deductively derived, subcodes and themes within each domain were developed inductively through close reading of the data. This allowed for the integration of participants’ narratives and perspectives while maintaining alignment with the food environment framework. This process was applied to 1 workshop transcript, during which subcodes were identified under each domain. The finalized coding system was organized hierarchically, with 6 “core codes” corresponding to the food environment domains. Furthermore, a gender-specific coding layer was applied to differentiate responses and insights by gender. This coding system was then applied to all the data. Throughout the analysis, the coding system was adjusted to better capture data complexity and nuances, and memos were systematically used to document analytical reflections, coding decisions, and emerging patterns. The coded data were jointly reviewed by the research team, and the main findings for each site were extracted. Primary coding and memo development were led by 1 junior researcher. Each team member conducted a preliminary synthesis of findings within an assigned food environment domain and site. Throughout the process, all 4 researchers reviewed the codebook, analytic memos, and the respective data to ensure a shared understanding of the coding framework and synthesis of findings. Consensus was reached through an iterative discussion process that drew on the codes, original transcripts (both pretranslation and post-translation where applicable), and the research questions [[40], [41], [42]]. Codes and thematic summaries were refined as needed, as agreement was reached.

MAXQDA mixed-methods data analysis software version 24 (24.3.0) was used to support the analysis. Sample data adequacy was determined by the relevance, specificity, and richness of the data in relation to the study aims and participant autonomy, which is a key feature of participatory video methodology. A COnsolidated criteria for REporting Qualitative research (COREQ) checklist was completed for transparency in reporting (Supplementary Table 1). Ethical approval for this research was obtained from the institutional review board of Hanoi University of Public Health (332/2023/YTCC-HD3). Furthermore, authorization was obtained from the 2 high schools in the rural and peri-urban sites and the community health center in the urban site to conduct study activities on their premises and to engage their staff members and students.

## Results

Adolescents captured all 6 domains of the food environment in their videos: *1)* food availability and accessibility; *2)* food price and affordability; *3)* food outlet properties; *4)* food products; *5)* food marketing and messaging; and *6)* food desirability. The footage and group discussions showed where they obtain their food, the price they pay, the products they consume, and the marketing they encounter. Although scenes and narratives are often intertwined in these domains in ways that mirror lived realities, we report results by domain to aid analytical clarity.

### Food outlets

Adolescents’ videos captured a range of settings where food is obtained and/or consumed, including the home, school canteens, family-owned shops, street food stalls, local fast food and speciality restaurants, convenience stores, supermarkets, and wet markets. The outlets filmed differed slightly across the 3 sites (Table 1).

**TABLE 1:** Food outlet types captured or mentioned by the adolescents, by site.

Outlet type	Rural	Peri-urban	Urban
Home	✓	✓	✓
School canteen	✓	✓	✓
Family-owned shop^1^	✓	✓	
Street food stall^2^	✓		✓
Local fast food and speciality restaurant^3^	✓	✓	✓
Convenience store^4^			✓
Supermarket		✓	
Wet market^5^		✓	✓

1 Family-owned shop: one-stop shops selling a diverse range of packaged foods and essential household items in closed-off and permanent premises, with extensions that may offer fresh foods.

2 Street food stall: located in the same places every day during certain hours in outdoor areas or on sidewalks with some form of seating capacity.

3 Local fast food and speciality restaurants: (small) food service outlets offering a limited range of quick, ready-to-eat food, and specialty dishes. With indoor food preparation and indoor and/or outdoor seating capacity and/or take away options. Meals may be based on staple ingredients such as rice, noodles, or bread, among others.

4 Convenience store: small-scale (chain) retail outlets that operate for extended hours, offering a limited range of ready-to-eat foods, beverages, fresh and frozen foods, packaged goods, and essential household items.

5 Wet market: small-scale vendors with temporary stalls, usually in an open space, at a recurrent place and time, offering fresh foods.

Eating at home and at the school canteen was presented at all the study sites. Supermarkets and convenience stores were shown, respectively, only in the peri-urban and urban sites. Street food stalls only featured in rural and urban sites, whereas wet markets were mentioned in peri-urban and urban sites.

Across sites, school canteens offered both freshly prepared meals (e.g., steamed sticky rice “*xôi”* with various toppings; noodle soups like “*bún riêu”)* and a wide range of packaged, processed, and ultraprocessed foods (UPFs), including potato crisps, instant noodles, ice-cream, sugar-sweetened beverages, and energy drinks. Although prominent in the peri-urban site, the school canteen was less featured and reportedly less preferred in the urban site. This was attributed to the immediate proximity and the more diverse options available near the school gate, as illustrated by, “I do not like the school canteen. Let’s go out to eat.” Girls’ video, urban site; and “There are many food carts (near the school) selling items like steamed sticky rice, steamed buns, bread and a variety of other dishes. Today, my choice is the convenience store, which is just 5 m from school.” Girls’ video, urban site.

Videos from peri-urban and urban sites showed snacks, sugar-sweetened beverages, and other UPFs prominently displayed in convenience stores and supermarkets. In the rural site, family-owned shops near homes and schools also offered packaged, processed, and UPF products, along with fresh food products. Street food stalls offered ready-to-eat meals and snacks.

Most food outlets featured in the videos were described as open throughout the day (from early morning to late evening), providing adolescents with vast choices and flexibility. Adolescents across the 3 sites commented on the fresh, clean, and appealing environment of outlets leaning on the “modern” side, such as local fast food and speciality restaurants, convenience stores, and supermarkets, for example, “The shop is nicely decorated, creating a feeling of relaxation and comfort.” Girls’ video, rural site; and “The restaurant is clean, with air conditioning, and they provide free tea.” Boys’ discussion, urban site.

In the rural site, fresh foods like eggs, meat, fruit, and vegetables were shown to be mostly sourced from home gardens and family-owned shops. Meanwhile, in the urban areas, adolescents reported relying on wet markets, directly and indirectly through their parents’ shopping practices, to source fresh foods for home-cooked meals.

Across all sites, videos showed adolescents eating alone and together with peers, at home, in school canteens, and in other outlets. Although family meals were not filmed, adolescents reported having regular family dinners at home: “Most students tend to dine at home and rarely eat out in the evening.” Girls’ discussion, rural site; and “Dinner is usually at home with the family, but it depends on the day... Dinner might be outside if we have extra classes.” Boys’ discussion, urban site.

At the same time, boys discussed how the videomaking process may have influenced what was filmed and emphasized that they often eat in the school canteen and at home, and are less likely in other places: “Boys do not eat out as much.” “In fact, boys often eat at the canteen.” “What is different from the video with reality is that when filming it was easier for us [the boys] to film eating alone, but [in fact] we eat with our families most of the time.” Boys’ discussion, peri-urban site.

### Food products

Across all sites, the videos showed adolescents selecting and consuming a diverse range of foods, supplemented by additional items mentioned during the discussions. The videos showed a clear prominence of packaged, processed, and UPFs, often offered alongside fresh, locally prepared, and minimally processed foods. (Minimally processed foods are natural foods that have undergone preparation or preservation processes without fundamentally altering their composition [43]). The diversity of food products shown within a given type of food outlet was generally higher in the urban site than in the rural and peri-urban ones.

The most featured food groups included cereals and grains; meat and fish; eggs; milk and dairy products; and vegetables. Fruit consumption was mainly reflected in the rural and urban videos and discussions. Roots and tubers were only mentioned in the peri-urban site. Table 2 summarizes the food products that featured in the videos and discussions, according to food group and site.

**TABLE 2:** Main food products featured by food group and study site.

Food group	Rural (Moc Chau)	Peri-urban (Dong Anh)	Urban (Dong Da)
Cereals and grains	Rice, rice/wheat noodles (fresh/instant), wheat spaghetti, bread	Steamed buns, bread, breakfast cereal, noodles (instant/fresh), rice, rice paper	Noodles (instant/fresh), rice
Roots and tubers	—	Potatoes	—
Meat and fish	Beef, chicken meat and feet, meat/fish balls, meat skewers, pork, sausages	Chicken meat and feet, duck, meat/fish balls, pork, sausages	Beef, crab, fish cakes, offal, pork, sausages
Eggs	Eggs	Eggs	Eggs
Milk and dairy products	Milk	Milk, yogurt	Milk, yogurt
Legumes and legume products	Peanuts	Tofu	Tofu
Fruit	Bananas, grapefruit, longans, and pomegranates	Apples, green mangoes	Apples, bananas, grapes, papaya, pears, pineapple, and pomegranates
Vegetables	Cabbage, carrots, *chayote*, *katuk*, mustard greens, mushrooms, and onions	Carrots, bean sprouts, and lettuce	Carrots, celery, cucumber, green leafy vegetables, jute leaves, lettuce, mustard greens, onions, and spinach
Other foods	Biscuits, potato crisps, and popcorn	Cakes, custard, and pudding	Potato crisps
Drinks	Chocolate-flavored milk, energy drinks, carbonated drinks, fruit juices, and water	Canned coffee, carbonated drinks, chocolate milk, energy drinks, flavored iced tea, and flavored malt drinks	Energy drinks, flavored iced tea, carbonated drinks, and sweetened milk

Minimally processed foods included: *1*) traditional rice or fresh noodle dishes (like *phở*—noodle soup; *bún riêu*—crab-based noodle soup with meatballs, tofu, bean sprouts, spring onions and tomato; *xôi*—steamed sticky rice; *cơm rang dưa bò*—fried rice with beef and pickled mustard greens); *2*) soups and sauces (like *canh cua rau đay*—crab meat and vegetable broth with jute leaves; *canh rau muống luộc*—morning spinach soup, *đậu sốt cà chua*—tofu in tomato sauce); *3*) sandwiches (like *bánh mì*—baguette with various savory fillings); *4*) steamed buns (*bánh bao*); and *5*) fried banana fritters (*bánh chuối*).

Packaged, processed, and UPFs included meats like sausages, meat/fish balls, dried beef, chicken feet, and pork floss; various types and brands of instant noodles; savory potato, wheat, or tapioca snacks and chips; and sweet pastries and biscuits. Beverages ranged from fresh fruit juices and fruit-flavored drinks, milk-based beverages (like sweetened milk, yogurt-based drinks, chocolate-flavored malt drinks), sweetened teas (like iced tea, bubble tea, and bottled flavored teas), coffee and coffee beverages (canned coffee), to soft drinks (carbonated) and energy drinks. It is worth noting that, during the video screening discussions, the adolescents remarked on the omission of water in their videos.

Across the 3 sites, the adolescents described having access to “unlimited” food options, with UPFs noted as a particularly popular choice for snacks. Quick and easy-to-make and -eat foods were reported as appealing, especially during school breaks, when time is constrained, or when preparing food themselves. This was more evident in the peri-urban and urban sites. For example, “Because I have to eat at 6 am in the morning to get to school on time, the food outlets aren't open yet, so I have no other choice, so I just eat instant noodles.” [Facilitator: If you had a choice, what would you choose?] Response: Bánh mì (baguette with various savory fillings) near my house. I make nước mật ong (honey water) myself.” Girls’ discussion, urban site.

Adolescents also noted that food preferences and choices were influenced by what was considered popular or trendy, recommended by peers, featured on social media or in advertisements, or with attractive packaging. As illustrated by “This is the new brand that I’ve heard of (instant noodles). I’ve been searching for it for a long time.” Girls’ video, rural site. Also, “The (fresh) noodles here (at the canteen) are very delicious, and it’s also close to the football field. ... This bowl has a lot of toppings. … Well, it’s delicious, I admit it’s as delicious as rumored. … Wow, the noodles taste great, I feel like it will make me full till the afternoon.” Boys’ video, peri-urban site.

The videos and discussions also showed a clear appreciation for and consumption of local and traditional foods as well as home-cooked meals, which were valued alongside new, trendy, or popular options and processed foods. “Home-cooked food is perfect.” Girls’ video, urban site; “Dinner at home is nutritious and diverse, offering delicious taste and flavor, while providing a great source of energy for studying.” Girls’ video, peri-urban site.

The food products’ sensory appeal was considered important, with adolescents frequently mentioning the taste, texture, appearance, and freshness of food products. “The food is freshly prepared after customers place their orders. Therefore, it is still warm and crunchy when served.” Girls’ video, peri-urban site.

In addition to taste, adolescents mentioned the perceived nutritional value and health benefits of foods, although their emphasis and understanding varied, including some misconceptions, and thus revealed uneven levels of nutrition literacy. Meat was considered an essential part of a meal (all sites); home-cooked meals were considered more nutritious and safer (urban and rural sites); fruit, vegetables, meat, and milk were highlighted as important foods (urban site); and protein-rich foods like meat, eggs, and tofu were considered beneficial for satisfaction and strength (peri-urban site). Some of these perceptions are illustrated by the following comments:

“A nutritious lunch—a full bowl of bún riêu (crab-based noodle soup), a bit oily, but delicious, with lots of toppings. Soft drinks provide essential minerals. All set for the day.” Boys’ discussion, urban site.

“Meat (pork) contains lots of vitamins…. Chayote also contains lots of nutrients that help rejuvenate the body, are a source of antioxidants and reduce stress.” Boys’ video, rural site.

“Morning spinach soup helps clear heat, detoxify and is delicious.” Girls’ video, rural site.

“In the bowl of instant noodles, there are sausages and meat skewers, which are delicious and contain lots of fat and protein, and there are vegetables, which provide good fiber. Overall, this bowl of noodles is full of nutrition.” Boys’ video, rural site.

“Today my main dish (for breakfast) is steamed Gac fruit sticky rice, … containing lots of starch, vitamins and nutrients, together with a sausage and a meat skewer… I find a breakfast like this very nutritious and affordable for students.” Boys’ video, rural site.

Across all sites, adolescents’ remarks considered sweetened foods and beverages good and nutritious, especially in the urban and peri-urban sites. This included chocolate-flavored malt drinks, yogurt, breakfast cereal, milk, fruit juice, and caffeinated drinks. “This energy drink is really delicious. When I am tired, drinking this helps.” Moc Chau girls’ video; and Person 1: “If you drink this can of coffee, how can you be full?” Person 2: “Coffee is nutritious and keeps me awake. It’s just 10,000 Dong for a can, so it’s cheap.” Boys’ video, peri-urban site.

### Food prices and affordability

The price of foods featured in the videos ranged from Vietnamese Dong (VND) 5000 (US$0.20) for instant noodles to VND 45,000 (US$1.80) for a full restaurant meal. Table 3 presents the cost of meals featured in the videos. Regardless of the site, processed and UPFs and beverages were considered affordable. In the peri-urban site, food prices seemed to be a critical factor when making food choices, often opting for the cheaper option. Although more expensive than packaged snacks (VND 5000–10,000/US$0.20–0.40), some food options were regarded as affordable, for example, VND 20,000 (US$0.80) for steamed sticky rice with various toppings of fried or processed meats (*xôi*). For example, “We chose steamed bun (bánh bao) and iced tea for the meal in the school canteen. Because we think this was a very convenient and quick food for students. The price was also affordable (total VND 20,000/US$0.80).” Girls’ discussion, peri-urban site.

**TABLE 3:** Meal prices per individual, according to the outlet and the site (in Vietnamese Dong)^1^.

Site	Rural	Peri-urban	Urban
Food outlets			
Steamed sticky rice, sausage, and meat skewer (pork)	20,000	—	20,000
Steamed sticky rice, sausages, fried eggs, and bottled tea	37,500	—	—
Rolls, potato fries, and mixed rice papers	—	20,000	—
Shredded dried beef, spicy noodles, fried chicken, fermented pork roll, pomegranate juice, and milkshake^2^	—	—	87,500
Spicy noodles with beef, ribs, mushrooms, and vegetables	—	—	40,000
Rice, tofu, pork ribs, chayote, and soup	—	—	40,000
Mixed instant noodles and bottled tea	28,000	—	—
Mixed fresh noodles	40,000	—	—
Crab-based soup noodle dish	—	—	15,000–30,000
Noodles with beef sausage	35,000	—	—
Noodles with beef and pork	45,000	—	—
Potato fries	15,000	—	—
Crab fresh noodles/pork rolls fresh noodles	20,000–30,000	—	—
Home-cooked meals^3^			
Spring rolls, biscuits	—	—	45,000
Instant noodles with pork balls	5000	—	—
Instant noodles	10,000	—	—
Spaghetti with chicken feet	—	—	40,000
Mixed instant noodles	10,000	—	—
School cafeteria and canteen			
Steamed sticky rice with pork floss	7000	—	7000
Rice, vegetables, vegetable soup, tofu, pork ribs	40,000	—	—
Pâté sandwich	10,000	—	—
Steamed sticky rice, processed meat, and pork floss	—	20,000	—
Steamed buns with meat and cheese	—	15,000	—
Snacks			
Steamed buns with meat	—	10,000	—
Potato crisps	20,000	—	20,000
Banana fritters	10,000	—	8000
*Que cay* (wheat/tapioca starch snack)	—	4500	—
Popcorn	—	—	5000
Drinks			
Energy drink	10,000		5000–10,000
Canned coffee	—	10,000	—
Flavored tea	10,000	5000	5000
Flavored drink	—	10,000	—
Single food item (i.e., ingredient for preparing a meal)			
Eggs (10 pieces)	—	—	30,000
Malabar spinach (bunch)	—	—	6000
Pork (to make spring rolls)	—1	—	15,000

During data collection, US$1 was equivalent to Vietnamese Dong 25,270.

1 The table only includes meals where the price was clearly shown or stated in the videos and discussions.

2 For some expenses, it is unclear how many adolescents were sharing the meal, so “—” is added instead.

3 For home-cooked meals, some ingredients such as vegetables, eggs, pork rolls, etc., were already available, and the costs were not mentioned, so the actual cost of those meals could not be accurately captured.

In the urban site, a preference for inexpensive and satisfying options was evident. At the same time, the videos showed that full meals (e.g., rice with tofu, pork ribs, chayote, and soup) were available and selected for a total cost of VND 40,000 (US$1.60), although they later reflected that this was “a bit expensive.” [Facilitator: “Bún riêu (crab-based soup noodle) for lunch, is that expensive?”] “It’s normal and suitable given the market prices. It’s affordable for students.” Boys’ discussion, urban site.

### Food messaging

Across the sites, videos captured various food-related messaging and marketing cues, including posters and branded materials (fridges, menus, packages, etc.), and advertisements and messaging on social media platforms. Food marketing largely featured processed and UPFs, especially sweetened beverages and instant noodles. This marketing was also prominent in canteens and restaurants in the rural and peri-urban sites. Across the 3 sites, adolescents were active users of social media platforms. In the peri-urban and urban sites, they identified family, school lessons, and social media as common sources of food-related information. For example, an advert for milk is shown on TikTok. Person 1: “Oh, the milk looks so delicious, I’ll have to tell my mom to buy it tomorrow.” The following morning*,* Person 2 asks*:* “Why have you chosen this for breakfast?” Person 1: “I have read that eating cereal with milk in the morning is very good for health.” Girls’ video, peri-urban site. Another example, “I watched this advert a couple of days ago and now I would like to eat it (black bean noodles, 10,000VND). I will go and buy it in [a convenience store], which is 5 min from my house. The price is quite reasonable.” Girls’ video, urban site.

### Interactions within the food environment

Across the sites, the videos showed adolescents preparing meals, either using ingredients they had purchased themselves or selecting from those available at home. At home, distinct roles were described; parents were identified as nutritional gatekeepers, controlling food provision and purchases; the adolescents acknowledged their own influence in making decisions about food options for different meals, taking on specific cooking tasks, or being responsible for younger siblings’ meals. Illustrations of this include:

“My mom went to the market. My mom decides what we eat; she often goes to Ngo Sy Lien market.” Boys’ video, Urban site.

“The food is chosen by my parents, based on my preferences, but also based on my parents’ preferences. They try to make it balanced for both.” Girls’ discussion, rural site.

“My mom buys ingredients and keeps them in the fridge, so I take them out and cook for myself. I prepare my own breakfast and also cook my own lunch.” Boys’ discussion, urban site.

“Most of the time, our parents cook lunch for us. On days when we get home early, we cook for ourselves.” Girls’ discussion, rural site.

The data presented indicated 5 main mealtimes: breakfast, lunch, afternoon meals, dinner, and late-night meals, as well as snacks. Late-night meals or snacks featured in 4 videos by both the boys and girls in the rural and urban sites, and were also mentioned in the discussions in all sites. Time was a key factor and was linked to reports of skipping breakfast across all sites. Some of the adolescents adapted by purchasing quick or ready-to-eat foods to fit their limited time: “Some days I have breakfast, other days I don’t. I will eat breakfast at school if I go to school or at home on my days off.” Boys’ discussion, urban site. “Some individuals may choose to eat at home or skip breakfast.” Girls’ discussion, rural site. “They (adolescents) have breakfast at the canteen.” “They can also have breakfast at home.” “They (adolescents/girls) don’t often have breakfast.” Girls’ discussion, peri-urban site.

## Discussion

Our study results indicate that adolescents’ food outlet and product choices were shaped primarily by affordability, proximity to school or home, and time constraints, making convenience and quick, ready-to-eat options particularly salient. Sensory appeal and the diversity of available food items also played a significant role, as well as the appeal of novel, trendy, popular, and familiar foods, suggesting context-dependent decision-making rather than fixed, internally consistent preferences and practices. This section discusses the interrelated factors influencing adolescents’ food choices across study sites, examining the accessibility, affordability, and appeal of both fresh and minimally processed foods, as well as UPFs in home and school food environments. It also explores the food messaging, norms, and peer and social media influences that adolescents navigate when making decisions, and considers the capacities and actions needed to support healthy diets and dietary choices.

Across sites, home and school food environments emerged as the primary settings where adolescents obtain and consume food. However, the mix of freshly prepared dishes and readily available UPFs, within and around the schools, suggests that school-focused interventions must extend beyond canteens to be effective. This aligns with mixed evidence on canteen use and gendered priorities [[44], [45], [46]] and our findings, where canteens were less frequently depicted in the urban and rural sites (especially in the boys’ videos). Because adolescents consider multiple factors when deciding on where and what to eat, the relative importance of factors may differ by gender. For example, although boys more frequently referred to portion size, girls highlighted food quality and social context. The motivations underpinning these gendered concerns are not completely clear in this context and signal the value of gender-responsive and context-specific approaches that address the diverse motivations underlying adolescents’ food choices.

Within the school food environment, it is widely recognized that improving the accessibility and appeal of nutritious foods and empowering adolescents to make healthy food choices to enhance the quality of their diets is key to promoting healthy diets. In Brazil, this was achieved through free and healthy public-school meals, working together with the adolescents to recognize their autonomy and leveraging their agency [14,47]. Furthermore, public policy or local government measures are needed to limit access to unhealthy foods, which, according to our results, were frequently sold alongside healthy options. These could include the development and enforcement of frameworks that establish nutritional criteria for school meals, and those that regulate foods and beverages available within and around school premises; criteria that limit the availability of foods that are high in sugar, salt, and fat, and that increase the availability and affordability of healthier options [48,49].

Affordability also played a role in decision-making and appeared to be especially salient in the urban study site. Consistent with evidence reported over 15 y ago, this confirms and highlights the ongoing relevance of affordability in adolescents’ food choices [21,50]. Food safety and convenience are also important factors that hinder or support out-of-home eating among adolescents [50]. To effectively contribute to improved nutrition outcomes, regulatory frameworks that ensure food environments are both nutrition-sensitive and culturally appropriate are needed [51].

Adolescents not only had access to a wide range of processed and UPFs but were also highly exposed to food-related information and trends on digital platforms, which strongly influenced their food choices. Such messaging often portrays less nutritious foods as convenient, desirable, and emotionally rewarding, reinforcing preferences for processed and UPFs. Social media food marketing is particularly powerful and persuasive; advertisements are often disguised as entertainment, influencer content, or posts from friends, making them harder to recognize as advertising [52]. Further evidence shows a small yet consistent association between media food marketing and adolescents’ eating outcomes. Exposure to these factors increases brand recall and fosters a more favorable attitude toward unhealthy foods—early mechanisms that can translate into consumption [52,53]. Additionally, viewing social media food advertisements while actively engaging with such content (liking, sharing, or following) is linked to higher intake of unhealthy beverages, which further increases the likelihood of consuming unhealthy foods and drinks, with stronger effects among frequent engagers [54]. Similar effects on food requests, choices, and intake have been reported for “advergaming”—video games developed to promote a product, brand, or service [55].

These key aspects call for coordinated regulatory and programmatic action that encompasses food marketing, and not simply action toward improving the availability and accessibility of healthy compared with unhealthy food within the school and immediate food environment. For example, incorporating food literacy into the school curricula can strengthen adolescents’ capacity to critically engage with nutrition information [56,57], and countermarketing campaigns to reduce the effects of persuasive advertising [52]. Combining complementary regulatory measures for unhealthy food advertising in and around schools and on social media [14] and promoting healthy food options through social marketing can support healthier choices. In fact, only 28% of 193 UN member countries have national policies restricting food marketing and/or competitive food sales in schools, and only 5 have extended the restriction beyond the school grounds [48], highlighting a major policy gap, especially in LMICs. A deeper understanding of the reach and impact of the different food-marketing techniques on adolescents’ knowledge, preferences, intentions, and consumption patterns is essential to further inform effective policy and intervention strategies.

With exposure to nutrition information from various sources, including family, school, peers, private-sector advertising, and social media, adolescents need to apply critical thinking and food literacy skills to effectively navigate food choice and purchase. Although our results show the adolescents’ limited engagement with food labels as part of the study, enhancing their ability to critically analyze and use the available nutrition information could significantly support healthier food choices. Studies report that adolescents lack the skills to effectively understand nutrition labels and use food labeling information satisfactorily, with some perceiving nutrition labels as too complex [58,59]. Improving food-label literacy has been shown to impact knowledge, attitudes, and the ability to identify healthier food options [59,60]. However, this impact is affected by the inconsistent clarity and completeness of nutrition information on food packaging. This was evident during the assessment of the nutrition labels of food products shown in the videos. This highlights the need for stronger enforcement of food labeling regulations and enhanced engagement with value-chain actors to ensure that nutrition information is accurate, consistently presented, transparent, and understandable.

A wide range of food outlets and food options were available and accessible to the adolescents at each site, providing autonomy, flexibility, and diversity to their food choices. Although the adolescents valued these elements, these options also pose drawbacks, as the combination of diverse options and varying levels of nutrition knowledge may not translate into healthy choices. Our data indicate uneven nutrition literacy among adolescents, including misconceptions about which foods are healthy. Even when adolescents can identify healthier compared with less healthy foods, the idea of being healthy alone does not always motivate their choices [14,61]. Consequently, nutrition education and literacy alone may be insufficient to promote healthy choices. Strategies should foster intrinsic motivation by aligning healthy eating with adolescents’ interests, social dynamics, and lifestyle contexts. For example, integrating hands-on cooking workshops, social media campaigns featuring relatable role models, or peer-led initiatives promoting foods that are both tasty and healthy could make healthy diets more appealing. Experiential approaches with children and adolescents have shown potential to improve nutrition knowledge, skills, attitudes, and consumption behaviors [[62], [63], [64]].

Irregular meal patterns were observed and attributed to a lack of time or the unavailability of quick and affordable options at a given time. Disrupted meal patterns among adolescents have been previously reported and were noted to increase when adolescents were out of the home [13,65]. These irregularities may increase reliance on unhealthy convenience foods/snacks, further compromising diet quality. Irregular meal patterns have implications for both physical and mental health, with studies showing links between meal frequency, skipping meals, and snacking associated with negative metabolic and psychological outcomes [66,67]. This underscores the need for integrated strategies that promote regular meal patterns and provide accessible, affordable options throughout the day, both at home and in and around school. For example, coordinated efforts to optimize adolescents’ daily routines, timetables, and eating spaces, paired with nutrition education that incorporates “nudge-based” approaches and subtle behavioral cues, may enhance healthy food choices while preserving individual choice autonomy [68]. “Nudge” approaches involve structuring the presentation of choices, such as organizing, displaying, or framing options to make healthier food items easier to select, without restricting available options or substantially changing the economic incentives [69,70]. Evidence indicates that such nudge-based approaches can modestly improve food choice, especially when combined with structural and pricing changes [[68], [69], [70]].

Additionally, cultural norms and peer behavior play a significant role in shaping preferences. Sharing food, inviting others, and eating with peers and family were valued by the adolescents in our study. This aligns with evidence that food practices in adolescence are socially meaningful [14,71]. Roles in acquiring, preparing, and choosing food can serve as an expression of agency and support identity, belonging, and autonomy [14]. These dynamics suggest that adolescents can act as catalysts for change, advocating for healthier eating habits and influencing family food choices.

This study reflects the researcher’s interpretation of data derived from adolescents’ lived experiences. The participatory video process was followed by the codevelopment of community action plans by adolescents and adults, which included participatory analysis of the respective food environments, including reflecting on the videos produced by the adolescents. The process and outcomes of this codevelopment phase are reported separately in another publication under development.

In conclusion, despite the prominence of processed foods and UPFs in the food environments and diets, the home food environment and consuming traditional foods remained an important part of the adolescents’ diets. Family and school education played an important role in providing food and nutrition information, but their influence is likely limited compared with peers, social media, and advertising. The interplay between home and school food environments offers valuable opportunities to improve their access to and consumption of healthy foods. This can be achieved through implementing and enforcing regulations that limit the marketing and sale of unhealthy foods, as well as promoting nutritious options within and around schools. Additionally, integrated strategies in both settings can *1*) increase the availability of appealing, nutritious foods; *2*) strengthen the nutrition knowledge of adolescents, parents, and school staff to support healthy choices and meal preparation; and *3*) encourage adolescent-led nutrition education and communication efforts. Using digital tools, social marketing, and subtle behavioral nudges can further support and motivate healthier eating habits among adolescents.

Further research is needed to better understand the complex, rapidly evolving food environments that adolescents navigate in Vietnam. This should include an in-depth study of food-choice motivators across diverse contexts and evaluations of the effectiveness of nutrition and health nudges, and behavioral cues in these settings. It should also entail *1*) assessing the impact of current food-marketing trends on adolescent food preferences, and *2*) examining how adolescents’ agility and involvement can be leveraged to promote and demand healthy foods. Additionally, research should explore how adolescents can be meaningfully engaged as agents of change through participatory approaches that use and enhance their capacity, agency, and autonomy, empowering them to improve their diets and influence their food environments. The adolescents and videos in this study were a part of the codevelopment of community action plans, during which the enhanced capacity and agency of adolescents to engage in this process were evident and valuable. The codevelopment process and results are presented in a complementary paper under development.

### Limitations of the study

Study limitations that should be considered when interpreting these findings. A methodological consideration is that the data were collected at a single point in time, therefore not accounting for seasonal food availability variations, school calendars, and other time factors that may affect food environments and practices. Although the participatory videomaking approach enabled the generation of rich, contextually grounded insights, it inherently captured curated representations of daily life. Participants themselves noted that some video scenes did not reflect all local adolescents’ experiences; certain practices were not regular occurrences; and some aspects of their food environment were not fully presented in the videos. The presence of the research team and the filming process may also have influenced participants’ food choices and practices. Lastly, prices and other quantitative details observed in the videos were not collected systematically and should therefore be interpreted as illustrative rather than comprehensive.

Despite its limitations, this study makes a meaningful and original contribution. By using a participatory videomaking approach, it captures lived experiences and the experiential dimensions of food choice that are essential for designing culturally grounded and adolescent-centered interventions. What adolescents choose to film reveals their priorities, concerns, and aspirations, offering valuable insights into their behaviors and decision-making. In a rapidly transforming food system, even cross-sectional visual ethnographic snapshots provide evidence on emerging dynamics, including digitalization and the growing presence of UPFs. The study diversifies methodological approaches in food environment research, pushing boundaries for how adolescent food environments can be examined in LMIC contexts. Although the findings are analytically rather than statistically generalizable, they generate a conceptual understanding relevant to similar contexts and lay the groundwork for future mixed-methods research. Finally, the study offers credible, contextually rich, and actionable evidence that has already informed community action planning and provides practical entry points for policymakers and other stakeholders to understand the food environment, identify and prioritize challenges, and guide more effective and locally owned action.

## Author contributions

The authors’ responsibilities were as follows – DN, IJ, CT: conceptualized the research; DN, BE: developed the implementation plan; DN, BE, THTM, PTTT: conducted the research and analyzed the data; TTM: contributed to study implementation and reviewed the manuscript; AF-T: contributed to data synthesis; DN, AF-T, BE, IJ, CT: wrote the paper; DN: primary responsible for the final content; and all authors: read and approved the final manuscript.

## Data availability

Data described in the manuscript will be made available on request, pending application and approval.

## Funding

This work was implemented as part of the CGIAR Initiative on Sustainable Healthy Diets through Food Systems Transformations (SHiFT) and the CGIAR Better Diets for Nutrition (BDN) Science Program, which were carried out with support from funders through their contributions to the CGIAR Trust Fund: https://www.cgiar.org/funders/.

## Declaration of generative AI and AI-assisted technologies in the writing process

The author(s) declare that no generative AI or AI-assisted technologies were used in the writing of this manuscript.

## Conflicts of interest

CT reports that financial support was provided by CGIAR. All authors declare that they have no known competing financial interests or personal relationships that could have appeared to influence the work reported in this paper.
