# Supplementary material for: Adolescents’ Interactions with Their Food Environment in Vietnam: A Participatory Video-Based Study on Factors Shaping Dietary Behavior
Source: Curr Dev Nutr. 2026 Apr 25;10(6):107706. doi: 10.1016/j.cdnut.2026.107706 (PMC13207457; doi:10.1016/j.cdnut.2026.107706)
Supplement: Multimedia component 1 [file mmc1.docx]

**Adolescents’ interactions with their food environment in Vietnam: A participatory video-based study on factors shaping dietary behaviour**

Deborah Nabuuma^1^, Brice Even^2^, Thai Hang Thi Minh^2^, Pham Thi Thanh Thu^2^, Andrea Fongar-Tiralla^3^, Truong Tuyet Mai ^4^, Irmgard Jordan^5^ and Céline Termote^5^

**Supplementary table COREQ table.**

| **No** | **Item** | **Description** | **Page number** | **Notes** |
| --- | --- | --- | --- | --- |
| **Domain 1: Research team and reflexivity** | | |  |  |
|  | Personal Characteristics | |  |  |
| 1 | Interviewer/facilitator | Which author/s conducted the interview or focus group? | 27 |  |
| 2 | Credentials | What were the researcher’s credentials? E.g. PhD, MD | 10 | Level of credential reflected by junior and senior researcher sufficient for the publication |
| 3 | Occupation | What was their occupation at the time of the study? | NA | Reflected by the author affiliations |
| 4 | Gender | Was the researcher male or female? | NA | Reflected by the author contributions |
| 5 | Experience and training | What experience or training did the researcher have? | 10 |  |
|  | Relationship with participants | |  |  |
| 6 | Relationship established | Was a relationship established prior to study commencement? | 7-8 |  |
| 7 | Participant knowledge of the interviewer | What did the participants know about the researcher? e.g. personal goals, reasons for doing the research | 7-8 |  |
| 8 | Interviewer characteristics | What characteristics were reported about the interviewer/facilitator? e.g. Bias, assumptions, reasons and interests in the research topic | 8-9; 10 |  |
| **Domain 2: study design** | | |  |  |
|  | Theoretical framework | |  |  |
| 9 | Methodological orientation and theory | What methodological orientation was stated to underpin the study? e.g. grounded theory, discourse analysis, ethnography, phenomenology, content analysis | 9 |  |
|  | Participant selection | |  |  |
| 10 | Sampling | How were participants selected? e.g. purposive, convenience, consecutive, snowball | 7 |  |
| 11 | Method of approach | How were participants approached? e.g. face-to-face, telephone, mail, email | 7 | In-person via the Vietnam National Institute of Nutrition in collaboration with local authorities |
| 12 | Sample size | How many participants were in the study? | 7 | 26 boys and 26 girls |
| 13 | Non-participation  Setting | How many people refused to participate or dropped out? Reasons? | 7 | 1 participant declined and was replaced. No participant withdrew |
| 14 | Setting of data collection | Where was the data collected? e.g. home, clinic, workplace | 9 |  |
| 15 | Presence of non-participants | Was anyone else present besides the participants and researchers? | 9 |  |
| 16 | Description of sample | What are the important characteristics of the sample? e.g. demographic data, date | 7 |  |
|  | Data collection | |  |  |
| 17 | Interview guide | Were questions, prompts, guides provided by the authors? Was it pilot tested? | NA | Workshop and observation guides, page 8-9 |
| 18 | Repeat interviews | Were repeat interviews carried out? If yes, how many? | NA |  |
| 19 | Audio/visual recording | Did the research use audio or visual recording to collect the data? | 8-9 | Videos from the participants and audio recordings from the workshops |
| 20 | Field notes | Were field notes made during and/or after the interview or focus group? | 8 | Observation protocols |
| 21 | Duration | What was the duration of the interviews or focus group? | NA |  |
| 22 | Data saturation | Was data saturation discussed? | 11 |  |
| 23 | Transcripts returned | Were transcripts returned to participants for comment and/or correction? | NA | Participants reviewed the developed videos but did not review workshop transcripts |
| **Domain 3: analysis and findings** | | |  |  |
|  | Data analysis | |  |  |
| 24 | Number of data coders | How many data coders coded the data? | 10 |  |
| 25 | Description of the coding tree | Did authors provide a description of the coding tree? | 10 |  |
| 26 | Derivation of themes | Were themes identified in advance or derived from the data? | 10 |  |
| 27 | Software | What software, if applicable, was used to manage the data? | 11 |  |
| 28 | Participant checking | Did participants provide feedback on the findings? | NA | Participants reviewed the videos they developed, page 8 |
|  | Reporting | |  |  |
| 29 | Quotations presented | Were participant quotations presented to illustrate the themes / findings? Was each quotation identified? e.g. participant number |  | Yes |
| 30 | Data and findings consistent | Was there consistency between the data presented and the findings? |  | Yes |
| 31 | Clarity of major themes | Were major themes clearly presented in the findings? |  | Yes |
| 32 | Clarity of minor themes | Is there a description of diverse cases or discussion of minor themes? |  | Yes |
